# Disentangling dissociative and nondissociative reaction dynamics in molecular mutual neutralization reactions between CO^+^ and O^−^

**DOI:** 10.1073/pnas.2603388123

**Published:** 2026-07-14

**Authors:** Mathias Poline, Arnaud Dochain, Stefan Rosén, MingChao Ji, Peter Reinhed, Ansgar Simonsson, Henrik Cederquist, Henning Zettergren, Henning T. Schmidt, Mats Larsson, Shaun G. Ard, Nicholas S. Shuman, Albert A. Viggiano, Richard D. Thomas

**Affiliations:** ^a^ Department of Physics, Stockholm University, Stockholm SE-10691, Sweden; ^b^ Institute of Condensed Matter and Nanosciences, Université Catholique de Louvain, Louvain-la-Neuve B-1348, Belgium; ^c^ Space Vehicles Directorate, Air Force Research Laboratory, Kirtland Air Force Base, NM 87117

**Keywords:** mutual neutralization, planetary atmospheres, cometary comae, molecular reaction dynamics

## Abstract

We have studied the mutual neutralization reaction of CO^+^ with O^−^ at a collision energy of ≤0.1 eV under single-collision conditions. We observe both fully dissociative (31.5 ± 1.8 %) and nondissociative (68.5 ± 1.8 %) charge transfer, involving at least five different electronically excited states in CO. From product momentum analysis, we find that the dynamics are governed by electron transfer processes at large O^−^-CO^+^ separations and the competition between predissociation and radiative decay in CO Rydberg states. In the case of nondissociative charge transfer, for one of the reaction channels we observe strong vibrational excitation (4 ≤ v≤ 8) in the product CO molecule. These data are expected to be useful for modeling astrophysical and planetary atmospheric environments where CO^+^ and O^−^ are present.

## Significance

The products and dynamics in mutual neutralization (MN) of CO^+^ with O^−^ occurring in planetary atmospheres and cometary comae are unknown, accessible only through new approaches that unite merged ion beams with long-time storage and coincident product imaging under cryogenic conditions, as realized at the double electrostatic ion ring experiment (DESIREE) facility. Here, the authors reveal a two-step mechanism via intermediate Rydberg states which, dependent on Rydberg-valence interactions, leads to competition between nondissociative photon-stabilization (68.5%) and dissociation (31.5%), which are identified. This demonstrates the utility of DESIREE for MN reactions of simple molecular ions, allowing detailed reaction-dynamics information to be extracted, significantly advancing theory and models of MN involving small molecules, and therefore of considerable interest to the wider community.

Mutual neutralization (MN) is an important reaction in the chemistry of natural and man-made plasmas. Over the past decade, the combination of merged ion-beam techniques with coincident neutral product imaging has allowed detailed information on MN reactions involving two atomic ions to be obtained, see e.g., refs. 1–5. Integrating these techniques with the advantages offered by cryogenic ion storage capability, such as implemented at the double electrostatic ion ring experiment (DESIREE) facility (6, 7), opens up the possibility to finally elucidate MN reactions involving molecular ions. Storage of the ions ensures relaxation of infrared active internal modes of molecular ions (8), simplifying interpretation of the data and ensuring the results are both more relevant for the descriptions of the plasmas in which such ions are typically found. Only since 2024 has DESIREE made it possible to study MN involving molecular ions and thus able to reveal critical insights into: proton-vs electron-transfer dynamics (9, 10); the dissociation dynamics of highly excited neutral molecular states (11, 12); and the effects of vibrational energy on steering these dynamics (12). Such studies have now opened a new frontier into charge-neutralization reaction investigations, and finally provide high-quality quantum-state specific data for testing molecular collision theories, see, e.g., refs. 9–12.

One fundamental aspect of MN reactions involving a diatomic molecular cation neutralizing with an atomic anion relates to breaking the molecular bond. In the recombination reaction of the same molecular cation with a free electron, the neutral molecule always dissociates, hence the name traditionally given to this process: dissociative recombination (DR). The recent groundbreaking studies carried out at DESIREE on the MN of NO^+^ with O^−^ (11), and of ${\text{O}}_{2}^{+}$ with O^−^ (12), also report that MN is exclusively accompanied by dissociation of the molecule, and that this occurs via a two-step process involving electron capture into predissociative Rydberg states. In neither of these neutralizing processes was electron capture into states above the neutral bond dissociation energy which subsequently stabilize through fluorescence nor into states below this energy (which cannot dissociate) observed. The neutral molecules NO and O_2_ have 0 K dissociation energies of 6.50 eV and 5.12 eV, respectively, and their relatively weak bond strengths compared to the excitation energies of the electron capture states in these neutral molecules may be the driver behind their dissociation. We therefore turn our attention to the molecule with the strongest chemical bond known in nature, namely CO, which has a binding energy of 11.11 eV.

Due to its stability, CO is the second most abundant molecule in the Universe after H_2_ (13, 14), and through its rich spectroscopy (15, 16) it has been observed in a wide variety of diverse planetary (17, 18), cometary (15, 19, 20), stellar (21), interstellar (13, 22), and even exo-planetary (23) environments. In general, electronic spectra of CO-containing plasma includes well-studied fingerprints, giving names to several of the important bands (see, e.g., ref. 16). Photoprocessing of the ubiquitous CO makes CO^+^ an equally important diatomic molecular ion in the same environments (24–34). For examples, CO^+^ is found in the coma and tail regions of comets (25–27), and in the atmospheres of planets such as Mars (17, 35) and Venus (18, 36), its presence in the latter likely arising from photoionization induced fragmentation of the dominant molecular species, CO_2_.

Neutralization reactions involving CO^+^ ions are therefore extremely important in these environments (31, 35). Unfortunately, and especially in comparison to DR, MN processes are often overlooked in plasma models of these regions, partly due to the lack of high-quality experimental and theoretical data. In particular, in environments not in local thermodynamic equilibrium (non-LTE), the full ionic reaction scheme does not have time to develop, and anions can be present in significant densities, for example, in lighting-induced sprites (12, 37, 38). Studies of the Venusian atmosphere also report sprite activity, see, e.g., ref. 39, and, furthermore, show that atomic oxygen is a critical electron acceptor (40). Elsewhere, for example, significant O^−^ densities are observed in cometary comae (41). MN between CO^+^ and O^−^ is therefore expected to be relevant in these regions.

In the MN of CO^+^ with O^−^ at 0 eV collision energy, the following final sets of products are energetically allowed:

[1a] $${\text{CO}}^{+}{(}^{2}\text{Σ})+{\text{O}}^{-}{(}^{2}\text{P})\to {\text{CO}}^{*}{(}^{1,3}\text{Σ})+\text{O}\;0\geq \text{Δ}H\geq -12.55\;\text{eV},$$

[1b] $$\;\;\to \text{C}{(}^{3}\text{P})+\text{O}{(}^{3}\text{P})+\text{O}{(}^{3}\text{P})\;\text{Δ}H=-1.40\;\text{eV},$$

[1c] $$\;\;\to \text{C}{(}^{1}\text{D})+\text{O}{(}^{3}\text{P})+\text{O}{(}^{3}\text{P})\;\text{Δ}H=-0.13\;\text{eV},$$

where the quoted enthalpies correspond to the energy released in each reaction outcome, given that both reactants are in their ground state (42–44). Reaction (**1**a) represents all possible final states of MN resulting in stable CO^∗^, while Reactions (**1**b and **1**c) represent the only two possible final channels for MN accompanied by molecular dissociation. In Reaction (**1**b), all three atoms are formed in their ^3^P ground-state terms, whereas [**1**c] corresponds to the carbon atom being excited to ^1^D at 1.27 eV excitation energy. Higher excitation of the carbon atom or any excitation of oxygen is not energetically accessible. Finally, fine structure energy differences are negligible in the present context. Depending on the outcome of the reaction at 0 eV, [**1**a–**1**c], the products have potentially different effects on the plasma in which the parent ions are found. Products with large kinetic energies may collisionally excite other plasma constituents or quickly leave their environment, while the decay of electronically excited CO^∗^ by photon emission would result in key spectroscopic signatures, as typically observed in, for examples, planetary atmospheres (17, 18), and cometary comae (15, 20, 32, 33).

Here, we report that the MN of CO^+^ with O^−^ at low collision energies, $E_{c.m}\leq$ 0.1 eV, mainly produces (68.5 ± 1.8%) five different electronically excited states of CO together with a ground state oxygen atom, in competition with a weaker (31.5 ± 1.8%), fully dissociative channel leading to purely ground-state atomic fragments only. The CO^+^ + O^−^ MN reaction thus leads to a radically different set of outcomes than the related DR reaction (45) as well as to the MN reactions of NO^+^ + O^−^ (11) and ${\text{O}}_{2}^{+}$ + O^−^ (12) which only lead to complete dissociation of the diatomic molecule. From product-momentum analysis, we find that the dissociation is driven by a two-step mechanism involving intermediate Rydberg states in CO, similarly to the process governing the MN of NO^+^ and ${\text{O}}_{2}^{+}$ with O^−^ (11, 12). However, unlike those two reactions, we reveal that dissociation of the CO out from these intermediate states competes with its stabilization by fluorescence. Fluorescence stabilization of the different electronically excited states of CO observed in the MN reaction is expected to give rise to an emission spectrum consisting of a range of photon energies, mostly in the UV (100 to 400) nm, with a few visible (430 nm) and infrared photons (1,029 nm) also possible. Furthermore, we report strong vibrational excitation in one of the electronically excited CO product channels.

## Results

The DESIREE facility, data acquisition methods, and Monte Carlo simulations have been described in detail elsewhere (6, 7, 11, 46). Here, we only give a short description: briefly, the cations and anions are injected and stored in separate storage rings, and merged in a common section of biased drift tubes. Neutral products of the MN reaction fly straight to a triple-stack MCP-phosphor-screen based detector, where the resulting photons are detected by a position- and time- sensitive camera (TPX3CAM). Coincident-product imaging is employed to determine the kinetic-energy released to the reaction products on an event-by-event basis to generate a kinetic-energy release distribution. The measured energy corresponds to the sum of the negative of the enthalpy of the reaction, −ΔH, given in Eq. **1**; the collision energy, $E_{c.m.}$, of the two ions; the change in internal rotational and vibrational energy between the reactants and products $\Delta E_{v,J}$, i.e.,

[2] $$\begin{array}{c} E_{K_{f}}=-\Delta H+E_{c.m.}-\Delta E_{v,J}, \end{array}$$

where $\Delta E_{v,J}<0$ when the rovibrational excitation energy is higher in the reactants than in the products. Analysis of all coincidence events allows separate $E_{K_{f}}$ kinetic energy spectra to be obtained for the two-body and three-body data, which in general is expected to correspond to stabilization and fragmentation of neutral CO formed in the initial, electron transfer step of the MN reaction, respectively. The contribution of three-body events, due to the dissociation of the CO^∗^ molecule, to the two body-spectrum is due to the limited detection efficiency, and is inferred by randomly selecting two out of three events from the three-body data, see *SI Appendix* and refs. 11 and 12. From this, we determine that the mutual neutralization reaction populates both dissociative and nondissociative (stable CO) reaction channels. The corrected distributions are then evaluated and fit to Monte Carlo simulations of the different observed product channels in a model of the experiment setup taking into consideration the length of the interaction region and the finite sizes of the two ion-beams into account (11, 12).

### Kinetic Energy Release Spectra.

**Fig. 1.**
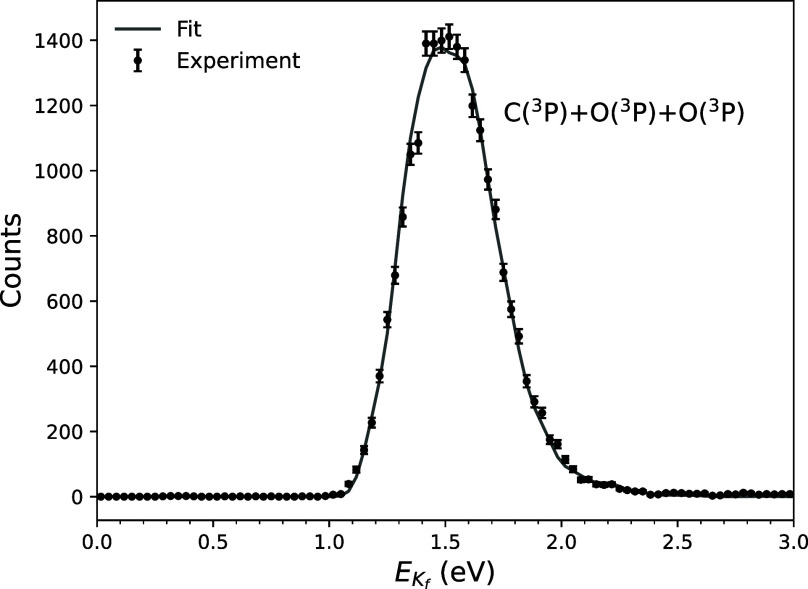
Coincident three-body total final kinetic energy, $E_{K_{f}}$ distribution at storage time t > 1 s. Experimental data are plotted as filled circles with statistical error bars. The simulated distribution is plotted as a gray line. The CO^+^ ions are assumed to be in their vibrational ground state, while their rotational energy distribution is described by a Boltzmann distribution with $T_{\mathrm{rot}}=1,300$ K.

The fully dissociative $E_{K_{f}}$ spectrum obtained from analysis of all three-particle coincidence events at a collision energy of $E_{c.m}\leq 0.1$ eV is shown in Fig. 1. Experimental data (filled circles) are plotted with statistical uncertainties. Data are consistent with the reaction proceeding via channel [**2**] only, i.e., ground-state C(^3^P) + O(^3^P) + O(^3^P) products are exclusively formed. There are no indications of significant signals below 1.0 eV. Given the production mechanism of CO^+^ from CO_2_ in the ion source, and in a similar approach to that carried out in analyzing the data obtained from the MN of O^−^ with NO^+^ and ${\text{O}}_{2}^{+}$ (11, 12), the CO^+^ ions are initially created with a distribution of vibrational energies, $E_{v}$, described by $T_{\mathrm{vib}}$ ≈ 3,000 K. Calculated vibrational radiative lifetimes for the CO^+^(${\text{X}}^{2}\Sigma$) ground state range from 128.5 ms for v=1 to 13.8 ms for v=9 (47) indicating a 99.9% population of v=0 after 1 s of ion storage. The data plotted in Fig. 1 were obtained after the ions had been stored for t > 1 s in order to ensure a cold vibrational population. Rotational relaxation lifetimes are typically a few orders of magnitude longer, see, e.g., ref. 8, and no significant rotational relaxation of the lower *J*-levels is expected. However, for convenience, the data are best fit in a Monte Carlo simulation assuming a Boltzmann rotational distribution described by $T_{\mathrm{rot}}$ ≈ 1,300 K.

**Fig. 2.**
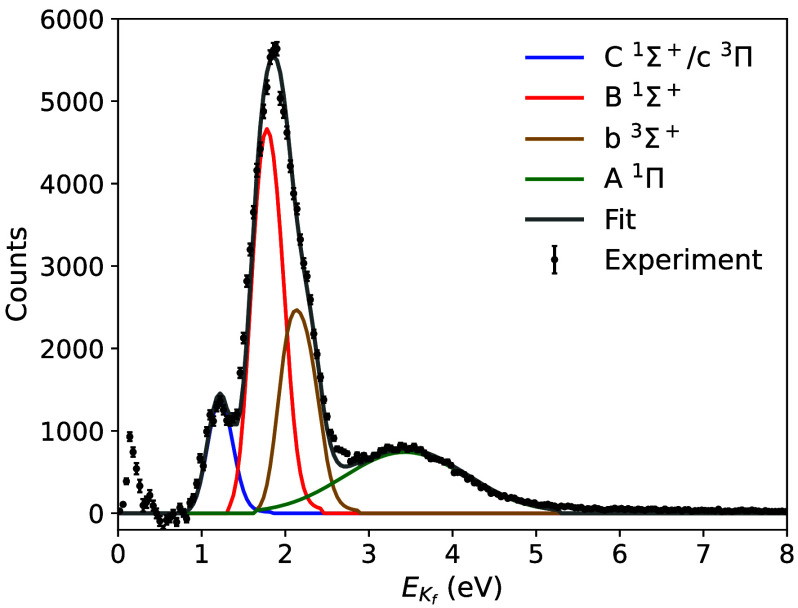
Coincident two-body total kinetic energy release $E_{K_{f}}$, distributions at storage time t > 1 s, with the false contribution from the dissociative channel due to the finite detector efficiency, subtracted (see text). Experimental data are plotted as filled circles with statistical error bars. The final fit of the simulated distributions is plotted as the gray line, with the indicated individual contributions shown by the colored lines.

The nondissociative two-body $E_{K_{f}}$ spectrum obtained under the same experimental conditions is shown in Fig. 2. Experimental data are plotted with statistical uncertainties, where contribution of the dissociative channel to the nondissociative spectrum has been subtracted (this process is described in *SI Appendix*). No signals are observed at larger $E_{K_{f}}$ values than ∼ 7 eV, indicating that the reaction does not lead to the formation of ground-state CO(${\text{X}}^{1}\Sigma$). After correction for the detector’s efficiency and acceptance, these data are found to account for 68.5 ± 1.8 % of the total MN signal, which puts the dissociative channel at 31.5 ± 1.8 %. This differs drastically from the MN of O^−^ with the two diatomic cations studied previously, NO^+^ and ${\text{O}}_{2}^{+}$, where dissociation completely dominates (11, 12). The structures observed in the two-body $E_{K_{f}}$ spectrum are consistent with the reaction producing several different electronically excited bound states of CO, in combination with a ground state O(^3^P) atom. The widths of the features in the spectrum differ with respect to each other, as well as to the single peak observed in the three-body fully dissociative $E_{K_{f}}$ spectrum (Fig. 1). This can be quantitatively understood from Eq. **2**. In a fully dissociative reaction, all the rotational energy in CO^+^ is converted into kinetic energy of the three atomic fragments, and $\Delta E\left(v=0,J\right)=0-E_{i}\left(v=0,J\right)$. Conversely, in the nondissociative two-body reactions, ΔE(v,J)∼0, unless the process drives the reaction to require a drastic change in rovibrational energy, which we will discuss later. Finally, a smaller feature at very low $E_{K_{f}}$ values is also observed. This is due to imperfect background removal, as is discussed in more detail in *SI Appendix*.

### Mutual Neutralization Reaction Dynamics.

For reactions which lead to complete dissociation, i.e., three atomic products, energy, and momentum conservation allows a wide range of break-up geometries. To understand how this dissociation occurs, the fragmentation dynamics in the three-body channel are investigated using Dalitz plot analysis (48) according to the standard generalized coordinates, $\eta_{1},\eta_{2}$, for an XY_2_ break-up (11, 49):

[3a] $$\eta_{1}=\frac{\sqrt{M/m_{C}}(E_{O1}-E_{O2})}{3E_{K_{f}}},$$

**Fig. 3.**
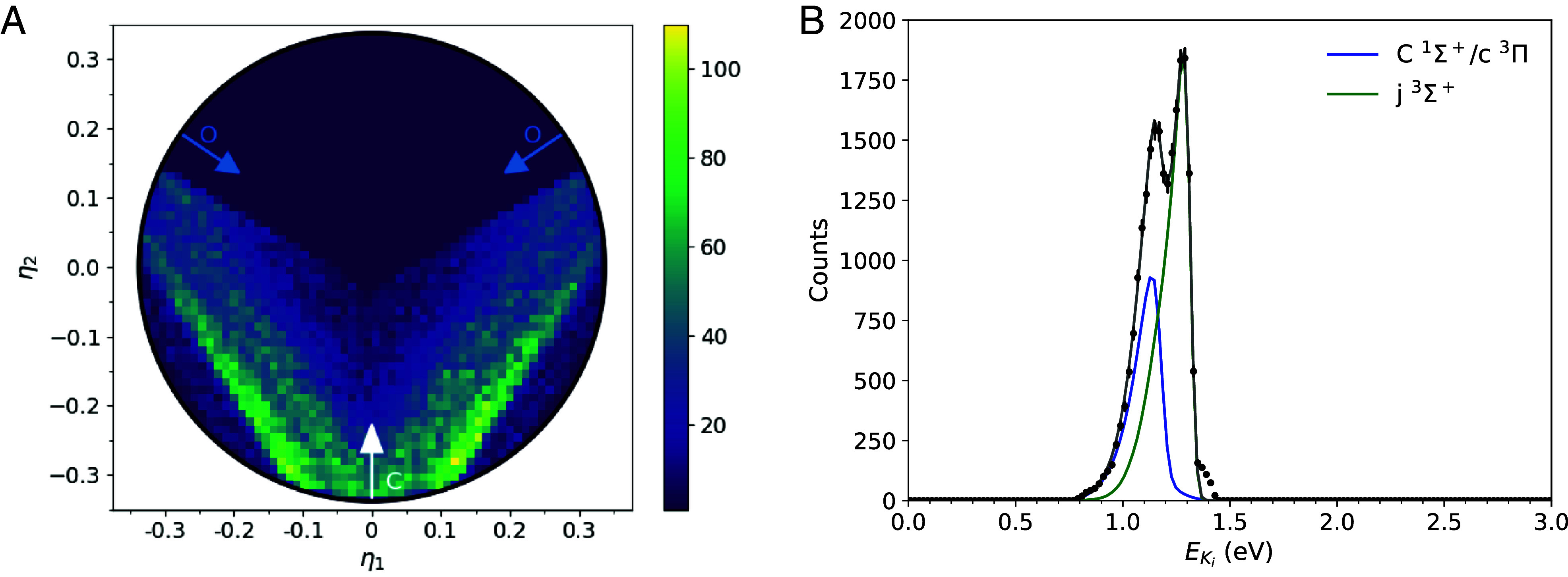
(*A*) Experimental Dalitz analysis of the fully dissociative C(^3^P) + O(^3^P) + O(^3^P) product channel; (*B*) the intermediate kinetic energy release, $E_{K_{i}}$, into the dissociative C(^3^P) + O(^3^P) + O(^3^P) channel.

[3b] $$\eta_{2}=\frac{(1+m_{C}/m_{O})E_{C}-E_{O1}-E_{O2}}{3E_{K_{f}}},$$

where $E_{C,O1,O2}$ are the kinetic energies of the three products, and $M,m_{C,O}$ the mass of the CO–O system and the individual atomic masses, respectively. Fig. 3 plots the fully dissociative data in these Dalitz coordinates. Since two of the three atoms are identical, the Dalitz plot has twofold symmetry, and the arrows shown in the figure denote the fraction of energy taken by each fragment, i.e., $\epsilon_{C,O}$. In this case, a technique was used to improve the particle identification procedure which artificially restricts particular $\eta_{1},\eta_{2}$ combinations (*SI Appendix*), resulting in empty regions in the plot. However, the main result resides in the lines of higher intensities perpendicular to the two oxygen atom axes. These features correspond to the situation where one of the oxygen atoms always takes a given fraction, $\epsilon_{O}$, of the available kinetic energy, $E_{K_{f}}$. This unambiguously requires that the state in CO which captures the electron is initially formed with a well-defined amount of internal energy, before subsequently dissociating. Although the observed structures are not completely resolved, they indicate that the observed three-body product channel is reached via two different intermediate states in CO.

The dynamics behind the fully dissociative reaction are thus similar to those observed in the MN of NO^+^ and ${\text{O}}_{2}^{+}$ with O^−^ (11, 12), which showed that the dissociation occurs via a two-step process. Given the nature of the reaction, further insight into the molecular dynamics can be retrieved if these two-step reactions are described as follows:

[4] $$\begin{array}{c} {\mathrm{CO}}^{+}+O^{-}\to {\mathrm{CO}}^{*}+O+E_{K_{i}}\to C+O+O+E_{K_{f}}. \end{array}$$

Assuming that no further energy is exchanged between the oxygen atom and the neutral molecule following the initial electron transfer step, the intermediate kinetic-energy release, $E_{K_{i}}$, can be evaluated from the energy of the neutralized atomic anion (12). Here, we apply a similar approach, where we instead use the experimentally measured fraction of energy given to the oxygen atom, $\epsilon_{O}$:

[5] $$\begin{array}{c} E_{K_{i}}=\left(M/m_{\mathrm{CO}}\right)\epsilon_{O}E_{K_{\mathrm{avg}}}, \end{array}$$

where $m_{\mathrm{CO}}$ is the mass of the CO molecule, and $E_{K_{\mathrm{avg}}}$ is the average value of the kinetic energy release, $E_{K_{f}}$, from the distribution plotted in Fig. 1. This approach effectively reduces the experimental broadening by minimizing the effect of the length of the interaction region (*Materials and Methods* and *SI Appendix*). Fig. 3 plots the intermediate kinetic-energy release, $E_{K_{i}}$, distribution obtained using this approach. Analysis of these data indicates two well-defined peaks, which correspond directly to the features observed in the Dalitz plot, and confirms the involvement of two different intermediate capture states which then couple to the same, final product channel.

## Discussion

Assuming negligible core mixing in CO, the electron must be captured into an electronic state with the same core configuration as the ion. The CO^+^ electronic ground-state (^2^Σ^+^) configuration is described by $1\sigma^{2}2\sigma^{2}3\sigma^{2}4\sigma^{2}1\pi^{4}5\sigma$. Electronic states of CO which satisfy this core electronic configuration are the ground state ${\text{X}}^{1}\Sigma^{+}$, and the a ^3^Π, A ^1^Π, b ^3^Σ^+^, B ^1^Σ^+^, j ^3^Σ^+^, c ^3^Π, C ^1^Σ^+^, and E ^1^Π excited states. Table 1 lists these electronic states together with their symmetry, the configuration of the outer electron, and the kinetic energy released on forming that particular CO state in v,J=0.

**Table 1.** List of the CO electronic states accessible in the MN of CO^+^ with O^−^: their symmetry; outer electron; predicted $E_{K_{f(i)}}$ values; and branching ratios in %

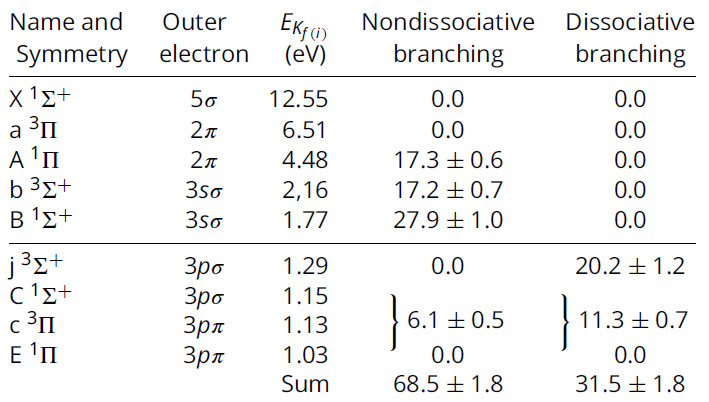

The horizontal dashed line separates electronic states which are below and above the C–O dissociation limit. The partial branching into individually observed product channels, as well as the sum of all dissociative and nondissociative final states are also listed.

**Fig. 4.**
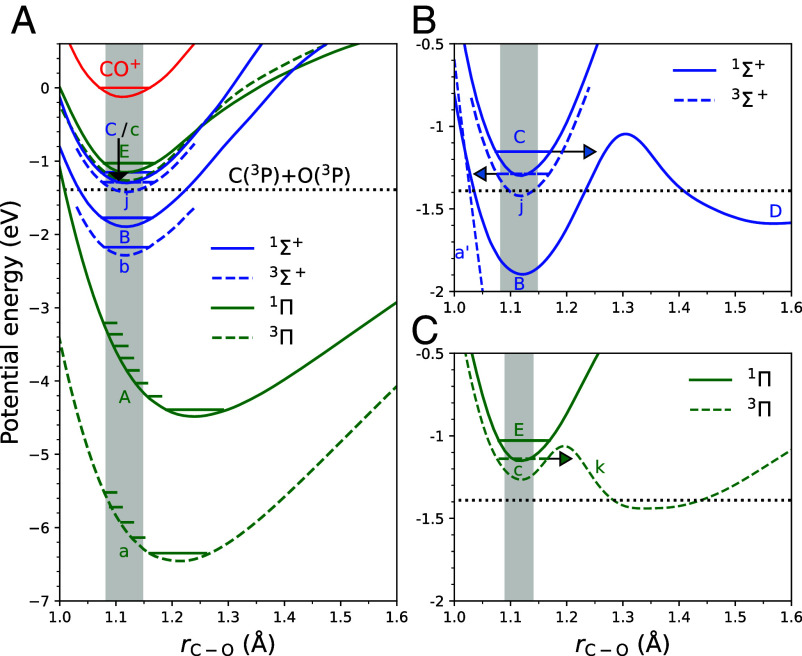
Potential energy curves for CO of relevance for the mutual neutralization of CO^+^ + O^−^ (see main text for discussion), adapted from refs. 50–52. The CO^+^ potential has been shifted by the electron affinity of O^−^ such that the energy of the intermediate CO^∗^ states directly corresponds to the relevant kinetic energy release. The gray band indicates the Franck–Condon region associated with a vertical electron-capture process. Panel (*A*) plots all the relevant states, and panels (*B*) and (*C*) focus on the states leading to dissociation with Σ− and Π− symmetry, respectively.

The potential energy curves (PECs) of these states, adapted from refs. 50–52, are plotted in Fig. 4. The CO^+^ (^2^Σ^+^) potential (red curve) has been shifted by the electron affinity of O^−^ such that the energy of the intermediate and final states directly corresponds to the associated kinetic energy release, $E_{K_{i}}$, $E_{K_{f}}$, respectively. The blue and green curves correspond to Σ− and Π−states, respectively, with the singlet and triplet spin symmetry of these states denoted by solid and dashed lines, respectively. Contributions from rotational and collision energy are neglected. If a vertical electron capture process is assumed, as inferred from our previous work, a Franck–Condon (FC) window region can be defined. The FC region indicates where on the PECs the electronically excited state would be formed following vertical electron capture, and is depicted by the vertical gray bands in Fig. 4.

Monte Carlo simulations were performed for each of these states, and were fit to the data presented in Fig. 2. The results of this fit reveals that the small peak centered at $E_{K_{f}}$≈ 1.25 eV is well described by capture into the c- and C-states, while the main peak at $E_{K_{f}}∼$ 2 eV correspond to capture into the b- and B- states. Given that the geometries of these four states do not differ much to that of the ion, these are assumed to be produced in the lowest vibrational level, depopulation of which is dominated by fluorescence (51, 53). The extremely broad feature, centered at ≈3.5 eV, has a completely different structure compared to the features at smaller $E_{K_{f}}$, indicating that the capture state is populated with a large range of vibrational energies. Analysis of the PECs shown in Fig. 4 indicates that the only state present in the appropriate potential energy region is the A ^1^Π state. As expected, this state does not have the appropriate Rydberg character, and intersects the FC window at its inner turning point at high vibrational levels (v=1 to v=8). Therefore, we assign this broad peak to capture into the A ^1^Π state which is populated with a large range of vibrational excitation, centered at v=4. Although the E ^1^Π and a ^3^Π states are accessible in an electron transfer process, they are not observed. We conclude that this is due to uncompetitive couplings between the ion pair curve and the relevant neutral PECs, which occur at extremely short and long CO-O separations, respectively.

For the fully dissociative channel, Monte Carlo simulations are performed within the framework of the Free Rotor model (*SI Appendix*). The results from fitting to the experimental data reveals that the two dominant features observed in Fig. 3 correspond to capture into the c-/C- and j-Rydberg states. Fig. 4 *B* and *C* plot the important PECs for the relevant Σ− and Π−states, respectively, as these couple out to different dissociative potentials. The j-state is strongly predissociated with an average lifetime of 4.6 ps (52). The exact predissociation pathways of this state are not known, but likely involve rotational coupling to predissociative vibrational levels in the a’-state, as indicated by the blue left-arrow. The situation is more complicated in the case of the C- and c-states. The C-state may couple to high vibrational levels of the B-state, which adiabatically couples to the dissociative D-state (Fig. 4*B*), while the c-state adiabatically couples directly to the dissociative k-state (Fig. 4*C*) (54). However, as can be seen from the right-pointing arrows, a small exit barrier is present along both of these dissociative pathways. Cacciani et al. report predissociation lifetimes from the C ^1^Σ^+^ state to be 1.78 ns for v=0 (51). However, they conclude that although this state predominantly decays radiatively, some low level of predissociation can not be excluded, given that different time domain measurements report on lifetimes ranging from 1.1 to 2.2 ns (55). This then results in the predissociation yield being anything between 0 and 28%. Experimental data for decay of the c-state is unfortunately sparse. In our data, we observe a peak at the kinetic energy release associated with the C-/c-states in both the nondissociative $E_{K_{f}}$ spectrum (Fig. 2) and the intermediate $E_{K_{i}}$ spectrum (Fig. 3) in the fully dissociative three-body channel. This indicates that one, or both, states must be partly predissociated. Unfortunately, given our current experimental resolution combined with the lack of spectroscopic data on these states, we can not draw more specific conclusions.

The remaining nondissociative channels all involve capture into excited states which lie below the dissociation limit. The production of ground-state CO (^1^Σ^+^) is not observed. As such, all of the nondissociative channels also are described by a two-step mechanism: capture into the intermediate excited state, followed by subsequent fluorescence down to the ground-state. *SI Appendix*, Table S1 lists potential fluorescence pathways from the observed electronically excited states of CO. Briefly, the emission spectrum is expected to consist of a range of photon energies, mostly in the UV (100 to 400) nm corresponding to the fourth positive band system (A ^1^Π → X ^1^Σ^+^), the Hopfield-Birge system (B ^1^Σ^+^ → X ^1^Σ^+^) and (C ^1^Σ^+^ → X ^1^Σ^+^), and the Cameron band (a ^3^Π → X ^1^Σ^+^). Fluorescence down from the A ^1^Π state to the ground X ^1^Σ^+^ state will give rise to a vibrational progression in the spectrum due to the fact that this state is initially formed with a highly vibrationally excited population centered about v=4.

## Conclusions

Mutual neutralization is an important reaction in the chemistry of natural and man-made cool plasmas. The combination of merged ion-beam storage techniques with coincident neutral product imaging has allowed detailed information on MN reactions involving molecular ions to be investigated in detail. Here, we directly measure the reaction products from the low-collision-energy mutual neutralization of vibrationally relaxed CO^+^ with O^−^. We demonstrate competition between fully dissociative and nondissociative electron charge transfer, where the reaction predominantly produces five different pairs of electronically excited states of CO, which we identify, together with a ground state oxygen atom, in competition with a weaker, fully dissociative channel leading to purely ground state atomic fragments. From product-momentum analysis, we conclude that the fully dissociative process occurs in a two-step mechanism via the intermediate j (^3^Σ^+^) and C(^1^Σ^+^)/c(^3^Π) Rydberg states in CO which couple out to the same final, dissociative state. However, we also reveal that this coupling out from the C(^1^Σ^+^)/c(^3^Π) states competes with nondissociative stabilization of the CO via fluorescence. Fluorescence of the different electronically excited states of CO observed in the nondissociative MN reactions are expected to give rise to an emission spectrum consisting of a range of photon energies, mostly in the UV (100 to 400) nm, with a few visible (430 nm) and infrared photons (1,029 nm) also possible. Furthermore, we also report that there is strong vibrational excitation in one of the electronically excited CO product channels, further complicating the fluorescence spectrum. In comparison with the fully dissociative outcomes from the related DR reaction of CO^+^ as well as the MN reactions of NO^+^ + O^−^ and ${\text{O}}_{2}^{+}$ + O^−^, the MN of CO^+^ + O^−^ thus leads to a radically different set of products. The present results are expected to be important for the development of models of astrophysical and planetary atmospheric environments where CO^+^ and O^−^ are present, and further drive development of theoretical frameworks for mutual neutralization reactions involving molecular ions in general.

## Materials and Methods

**Fig. 5.**
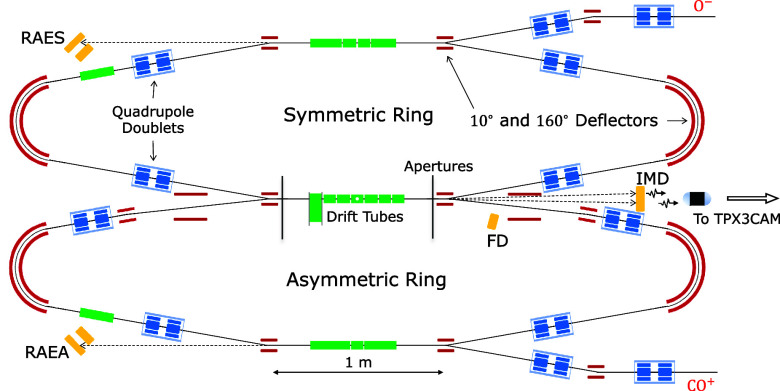
Schematic of the experimental setup at DESIREE. The two oppositely charged ion beams are injected and stored in their respective rings by the use of quadrupoles and deflectors. Between the two pick-up (PU) electrodes, they are merged together and interact freely. The neutrals formed in the MN reactions continue straight to the imaging detector (IMD).

The experiment was carried out at the double electrostatic ion-beam storage ring facility (DESIREE) located at Stockholm University. The facility is described in detail here (6, 7, 11, 12), and a schematic of the two rings and their common merging section is presented in Fig. 5. Briefly, carbon dioxide, CO_2_, was used as a source gas to produce the CO^+^ cation beam in an electron cyclotron resonance (ECR) ion source (Pantechnik Monogan). The CO^+^ ions were extracted and accelerated to a final beam energy of 17 keV, and then injected into the so-called asymmetric ring (Fig. 5). The O^−^ beam, produced by cesium sputtering of a SnO_2_ cathode (National Electrostatics Corp.), was extracted and accelerated to 8 keV and injected into the other ring.

During each orbit, the CO^+^ and O^−^ ion beams are overlapped in a common straight section of drift-tubes, before being demerged back into their respective rings. A voltage of 1,090 V was set on three of the tubes to accelerate and decelerate the anions and cations, respectively, in order to match their velocities and reach the lowest collision energy possible. At DESIREE, this is typically between 50 and 100 meV, due to the small angular spread of the beams. This is satisfactory, in regard to the environmental conditions being reproduced (between 300 and 1,000 K, corresponding to a collision energy of 30 to 130 meV). We note here that the MN cross section typically increases as the collision energy decreases, and that events from the biased region dominate as events from outside these regions are easily distinguished by their large time separations. For an MN event taking place at a distance *L* from a detector, with ions moving at speed *v* in the laboratory system, the kinetic energy of a product with mass $m_{i}$ in the center-of-mass system is

[6] $$\begin{array}{c} E_{i}=\frac{v^{2}}{2L^{2}}m_{i}{\vec{r_{i}}}^{2}, \end{array}$$

where $\vec{r_{i}}=\left(\Delta x_{i},\Delta y_{i},v\Delta t_{i}\right)$ is the position vector of the particle, relative to the center-of-mass of the products. Coincidence measurements allow to determine the number of products, i.e., i=2,3, and the sum of the kinetic energies of all of the products. This yields the final kinetic energy released in the reaction $E_{K_{f}}$, which is the sum of the reaction’s kinetic energy release $E_{K}$, the collision energy, $E_{c.m.}$, and the internal energy of the cation, $E_{v,J}$, in the case of three reaction products. For a three-body break-up, $E_{K_{f}}=E_{1}+E_{2}+E_{3}$, and the intermediate kinetic energy in the reaction $E_{K_{i}}$ is retrieved by momentum conservation from the kinetic energy of the neutralized anion. In this case it corresponds to the sum of the initial kinetic energy release $E_{K}$, the collision energy $E_{c.m.}$, and the difference in internal energy $\Delta E_{v,J}$, between the initial and intermediate state.

The neutral particle detection apparatus, located at about 1.77 m from the center of the selected interaction region, consists of a triple stack of 75 mm-diameter microchannel plates (MCPs) with a similar sized phosphor-screen anode. Light from the phosphor is coupled out from the cryogenic environment, and imaged onto an optical camera with a 256×256 pixel array (TPX3CAM, see, e.g., refs. 11, 12, 56, and 57). Each pixel in the camera is effectively its own photomultiplier (PMT) system, allowing for the time information of each event to be recorded. The positions of each neutral are retrieved from the centers of each group of illuminated pixels on the camera.

## Supplementary Material

Appendix 01 (PDF)

## Data Availability

All data underlying the figures are deposited in the following Zenodo repository (58) (https://doi.org/10.5281/zenodo.17980206). All other data are included in the manuscript and/or *SI Appendix*.
