# Supplementary material for: Disentangling dissociative and nondissociative reaction dynamics in molecular mutual neutralization reactions between CO+ and O−
Source: Proc Natl Acad Sci U S A. 2026 Jul 14;123(29):e2603388123. doi: 10.1073/pnas.2603388123 (PMC13389678; doi:10.1073/pnas.2603388123)
Supplement: Supplementary file 1 — Appendix 01 (PDF) [file pnas.2603388123.sapp.pdf]

Supporting Information for

Disentangling dissociative and non-dissociative reaction dynamics in molecular mutual neutralisation reactions between CO^{+} and O^{-}.

Mathias Poline, Arnaud Dochain, Stefan Rosén, MingChao Ji, Peter Reinhed, Ansgar Simonsson, Henrik Cederquist, Henning Zettergren, Henning T. Schmidt, Mats Larsson, Shaun G. Ard, Nicholas S. Shuman, Albert A. Viggiano, and Richard D. Thomas

Richard D. Thomas
E-mail: rdt@fysik.su.se

This PDF file includes:

Supporting text
Figs. S1 to S2
Table S1
SI References

Supporting Information Text

In order to evaluate the data, appropriate methods must be used to determine the momentum vectors and energies of the products, and Monte Carlo simulations of the particles trajectories must be implemented. The reaction can in principle lead to both two- and three-body products, and these are treated independently.

1.1 Two-body analysis.

In a two-body scenario, i.e reaction (1a) in the main manuscript, both products receives a fixed amount of energy, and the calculation of their final kinetic energy doesn't require identification of the products (see, e.g. (1)). The resultant experimental 2-body E_{K_f} spectrum for the MN of CO^+ with O^- is shown in Fig. S1, plotted as filled black circles with statistical error bars. As the MCP detection efficiency is not 100%, three-body events in which one particle is not detected, but which still satisfies centre-of-mass filtering, contribute to the 2-body E_{K_f} data. This contribution can be evaluated by randomly selecting two products out of the three from the three-body data presented in the main article in Fig. 1, and these data are indicated by the blue line shown here in Fig. S1. Comparison of these two datasets allows the true, two-body E_{K_f} spectra to be determined.

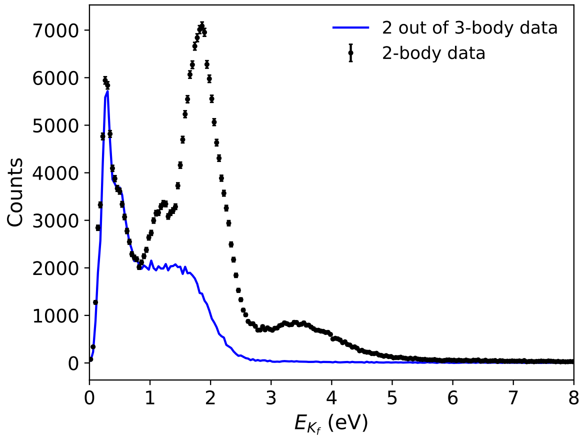

Fig. S1. E_{K_f} distributions for two-body coincidence data. The data indicated by the filled black circles shows the measured two-body data, and the blue line the contribution from three-body data where one neutral product is randomly removed.

After subtracting contributions from the three-body data, analysis of the resultant two-body E_{K_f} data plotted in Fig. S1 reveals distinct and significant structures. These features are consistent with the MN reaction producing several different electronically excited bound states of CO, but no ground-state CO, in combination with a ground state (^{3}P) oxygen atom, as well as a single, fully dissociative reaction channel. The minor feature at very low E_K values (< 1 eV) is due to to the 2-out-of-3 background subtraction does not perfectly describe the data, possibly due to differences in the real detection sensitivity for two and three products. Additionally, there may occur three-particle events from collisions at higher energies, where we then happen to detect the two atoms from the molecule. These events will come close in time, but will not be represented by the two-of-three data based on three-body events at E_{cm}=0. In addition, the two-body data likely contains some false coincidences at such small separations due to technical limitations in the imaging detector.

1.2 The free-rotor model in two-step reactions.

In the case of a dissociation via an intermediate state, the reaction can be described as follows:

$$CO^+ + O^- \rightarrow CO^* + O + E_{K_i} \rightarrow C + O + O + E_{K_f}, \qquad [1]$$

i.e., there is an intermediate kinetic energy release E_{K_i} which leaves CO in an excited state, and there is a subsequent, final kinetic energy release, E_{K_f}, for the complete process which includes the kinetic energy released in the dissociation of CO^*. Within the free-rotor model (2, 3), we assume that the neutralised anion no longer participates in the reaction after the initial electron-transfer step, i.e. no further energy is then exchanged between O and CO^*. The fraction of the available energy taken by the neutralised anion is then determined by the kinetic energy, E_{K_i}, released as the intermediate state CO^* is formed. As seen from the centre-of-mass of CO^*, the kinetic energies of the two atoms are well defined, and their momenta opposite. However, in the centre-of-mass of all three atoms, the lengths of the momentum vectors are in general different.

From conservation of energy and momentum, the kinetic energy released when the intermediate state is formed is determined by the kinetic energy, E_O, of the neutralised anion:

$$E_{K_i} = \frac{2m_O + m_C}{m_O + m_C} E_O \qquad [2]$$

However, in this particular reaction, given that only one three-body channel is populated, the resolution may be improved by using the fraction of energy of this atom instead:

$$E_{K_i} = \frac{2m_O + m_C}{m_O + m_C} \frac{E_O}{E_K} E_{K_{\text{avg}}} \qquad [3]$$

where $E_{K_{\text{avg}}}$ is the average final kinetic energy release of the three-body channel ($\sim$ 1.5 eV). Given that the measured values of E_O and E_{K_f} scale with the distance of the interaction region in the same way (see equation (6) in the main manuscript), the broadening due to the length of the interaction region is effectively eliminated. Here, given that two of the three particles have the same mass, specific selections are applied to aid the particular identification, based on the observed features in the Dalitz plot (see Fig. 3(a) in the main manuscript), which we will discuss in the next section.

1.3: Mass assignment.

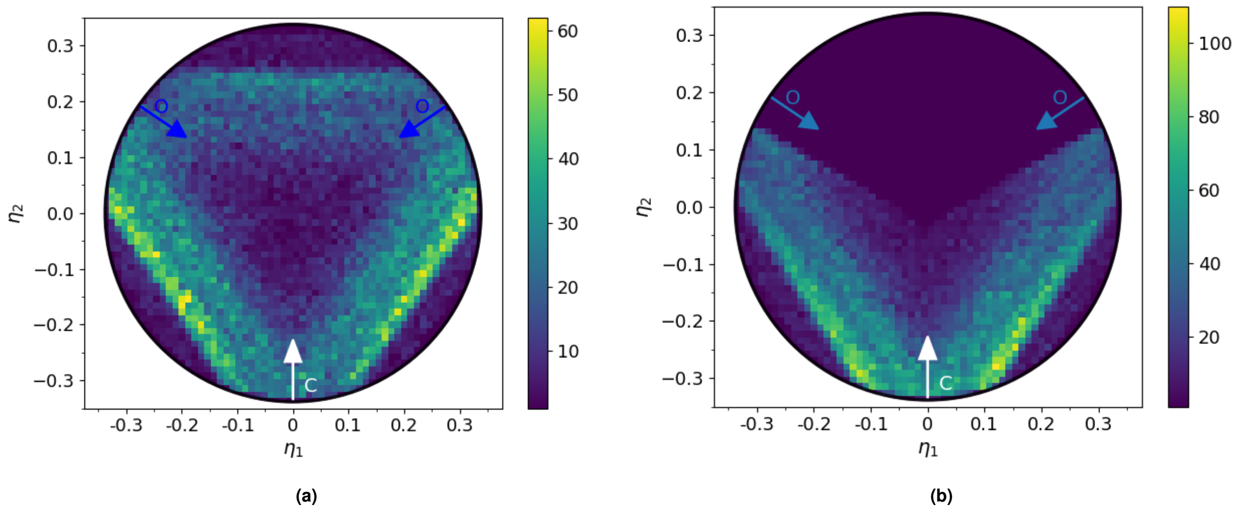

Fig. S2. Experimental Dalitz analysis of the fully dissociative C+O+O product channel with (a) standard mass assignment (b) additional correction.

The imaging detector is not mass-sensitive and the identity of each particle must be therefore deduced by other means. This is achieved by first determining the experimental (x,y) centre-of-mass of the two beams, and then, for each three-particle event, compute the three possible permutations of their centre-of-mass. The permutation being closest to the two beams' centre-of-mass is selected, allowing a mass to be assigned to each particle. However, given that the ion beams have a certain radius, there is an associated distribution of their collective centre-of-mass on the detector. As such, for certain events, the method can result in a misassignment of the particles. This can be seen in the Dalitz plot shown in Fig. S2a. Distinct areas of higher intensities can be observed, which indicates one of the three particle is taking a large fixed fraction of the available energy. This feature is three-fold symmetric, but of lesser intensity for the C atom, which is consistent with the expected probability of falsely assigning the C atom as an O atom. This misassignment can be corrected by assuming that the C atom can not take the largest fraction of energy in the process. Using this assumption in the data analysis retrieves the Dalitz plot shown in Fig. S2b, which is then used to retrieve the intermediate kinetic energy release E_{K_i} spectrum shown in the main manuscript.

1.4: Non-dissociative fluorescence pathways.

Fluorescence stabilisation of the different electronically excited states of CO observed in the MN reaction is expected to give rise to an emission spectrum consisting of a range of photon energies. Table S1 lists potential pathways by which the electronically excited states may fluoresce down to the ground-state. For the fluorescence photon energies, electronic state energies have been taken from ref. (4).

The (A $^1\Pi \rightarrow$ X $^1\Sigma^+$) transition is know as the fourth positive band system, and, together with transitions in the Hopfield-Birge system (B $^1\Sigma^+ \rightarrow$ X $^1\Sigma^+$) and (C $^1\Sigma^+ \rightarrow$ X $^1\Sigma^+$), and the Cameron Band (a $^3\Pi \rightarrow$ X $^1\Sigma^+$) would contribute to the main UV features in the emission spectrum, see e.g. (4). Fluorescence from the A $^1\Pi$ state directly down to the ground X $^1\Sigma^+$ state will give rise to a broad emission spectrum due to the fact that this state is populated with a large range of initial vibrational energies centred about $v = 4$. Fluorescence from the b $^3\Sigma^+$ state directly to the ground state is forbidden, and so likely occurs in two steps: first down to the a $^3\Pi$ state (the Third positive band) and then down to the ground state (the Cameron band), where both these transitions are also in the UV. Additional fluorescence pathways are also possible from the B $^1\Sigma^+$ state: first a transition down with a blue photon to the A $^1\Pi$ state (Ångström Band), followed by a (A $^1\Pi \rightarrow$ X $^1\Sigma^+$) fourth positive

Table S1. Potential fluorescence pathways from the electronically excited states of CO observed in the non-dissociative MN reactions of CO^+ + O^- at the given kinetic energy release, E_{K_f}. Solid horizontal lines separate the initial electronically excited states, while dashed horizontal lines separate different potential fluorescent pathways from the same initial state. Δv=0 transition wavelengths are given, unless otherwise stated, as are the names generally associated with these transitions

State	E_{K_f} (eV)	Branching (%)	Potential fluorescence scheme(s)	Wavelength (nm)	Notes
A $^1\Pi$	4.48	17.3±0.6	A $^1\Pi \rightarrow$ X $^1\Sigma^+$	130-160 (Δv=0-8)	Fourth positive band
b $^3\Sigma^+$	2.16	17.2±0.7	b $^3\Sigma^+ \rightarrow$ a $^3\Pi$,	283	Third positive band
			a $^3\Pi \rightarrow$ X $^1\Sigma^+$	208	Cameron band
B $^1\Sigma^+$	1.77	27.9±1.0	B $^1\Sigma^+ \rightarrow$ X $^1\Sigma^+$	114	Hopfield Birge band
			B $^1\Sigma^+ \rightarrow$ A $^1\Pi$,	430	Ångström Band,
			A $^1\Pi \rightarrow$ X $^1\Sigma^+$	155	Fourth positive band
C $^1\Sigma^+$	1.15	6.1±0.5 (shared with c $^3\Pi$)	C $^1\Sigma^+ \rightarrow$ X $^1\Sigma^+$	108	Hopfield Birge band
			C $^1\Sigma^+ \rightarrow$ A $^1\Pi$,	359	Herzberg band system
			A $^1\Pi \rightarrow$ X $^1\Sigma^+$	155	Fourth positive band
c $^3\Pi$	1.13		c $^3\Pi \rightarrow$ X $^1\Sigma^+$	107	
			c $^3\Pi \rightarrow$ a $^3\Pi$,	222	
			a $^3\Pi \rightarrow$ X $^1\Sigma^+$	208	Cameron band
			c $^3\Pi \rightarrow$ b $^3\Sigma^+$,	1029	
			b $^3\Sigma^+ \rightarrow$ a $^3\Pi$,	283	Third positive band
			a $^3\Pi \rightarrow$ X $^1\Sigma^+$	208	Cameron band

band system UV transition mentioned above. Similarly, for the C $^1\Sigma^+$ state: an initial transition down with a UV photon to the A $^1\Pi$ state (Herzberg Band), again followed by a (A $^1\Pi \rightarrow$ X $^1\Sigma^+$) fourth positive band system UV transition. Finally, multiple fluorescence pathways are possible from the c $^3\Pi$ state. A direct (c $^3\Pi \rightarrow$ X $^1\Sigma^+$) hard UV transition is least likely as this transition is reported to be extremely weak in absorption, see e.g. (5). A two-step process, with fluorescence down to the a $^3\Pi$ state, see e,g, (6) (c $^3\Pi \rightarrow$ a $^3\Pi$) followed by a transition down (the Cameron band) to the ground state (a $^3\Pi \rightarrow$ X $^1\Sigma$) likely dominates. Finally, a near infra-red transition between (c $^3\Pi \rightarrow$ b $^3\Sigma^+$) (7) would then couple to the ground state in two UV-photon steps as mentioned above: (b $^3\Sigma \rightarrow$ a $^3\Pi \rightarrow$ X $^1\Sigma^+$).

References

1. M Poline, et al., Mutual neutralisation of O^+ with O^-: investigation of the role of metastable ions in a combined experimental and theoretical study. *Phys. Chem. Chem. Phys.* **23**, 24607–24616 (2021).
2. A Hishikawa, H Hasegawa, K Yamanouchi, Sequential three-body Coulomb explosion of CS_2 in intense laser fields appearing in momentum correlation map. *Chem. Phys. Lett.* **361**, 245–250 (2002).
3. M Poline, et al., Mutual Neutralization of NO^+ with O^-. *Phys. Rev. Lett.* **132**, 023001 (2024).
4. M Khalil, et al., Theoretical investigation of the $A^1\Pi - X^1\Sigma^+$, $B^1\Sigma^+ - X^1\Sigma^+$, $C^1\Sigma^+ - X^1\Sigma^+$, and $E^1\Pi - X^1\Sigma^+$ transitions of the CO molecule. *Phys. Chem. Chem. Phys.* **27**, 2783–2801 (2025).
5. SG Tilford, Evidence for the Reclassification of the Upper State of the 3A Bands in the Spectrum of CO: The $c^3\Pi \leftarrow X^1\Sigma^+$ Transition. *The J. Chem. Phys.* **50**, 3126–3127 (1969).
6. R Hakalla, Analysis of the $c^3\Pi(v = 0, 1)$ State on the Basis of the 3A Band System in the $^{12}C^{16}O$, $^{13}C^{16}O$, and $^{14}C^{16}O$ Molecules. *J. Mol. Spectrosc.* **209**, 71–80 (2001).
7. I Dabrowski, M Vervloet, DC Wang, The $c^3\Pi$-$b^3\Sigma^+$ transition of CO near 8245 cm^{-1}. *Can. J. Phys.* **65**, 1171–1177 (1987).
